# Safety and diagnostic yield of transbronchial lung cryobiopsy in patients with interstitial lung disease and severe pulmonary function impairment

**DOI:** 10.1186/s12931-026-03957-z

**Published:** 2026-10-13

**Authors:** Christoph Lederer, Markus Polke, Janna Reinhard, Ralf Eberhardt, Katharina Buschulte, Monika Eichinger, Claus Peter Heußel, Enikő Fuchs, Konstantina Kontogianni, Michael Allgäuer, Felix J. Herth, Franziska C. Trudzinski

**Affiliations:** 1 Center for Interstitial and Rare Lung Diseases, Department of Pneumology and Critical Care Medicine, Thoraxklinik Heidelberg, University Hospital Heidelberg, Heidelberg, Germany; 2 Translational Lung Research Center Heidelberg (TLRC-H), German Center for Lung Research (DZL), Heidelberg, Germany; 3 Department of Pulmonary Medicine, Ruhrlandklinik University Hospital Essen, Essen, Germany; 4 Department of Pneumology and Intensive Care Medicine, Asklepios Klinik Barmbek, Hamburg, Germany; 5 Department of Diagnostic and Interventional Radiology with Nuclear Medicine, Thoraxklinik Heidelberg and Department of Diagnostic and Interventional Radiology, University Hospital Heidelberg, Heidelberg, Germany; 6 Department of Pathology, University Hospital Heidelberg, Heidelberg, Germany

**Keywords:** Interstitial lung disease (ILD), Transbronchial lung cryobiopsy (TBLC), Complications, Diagnostic yield, Severe pulmonary function impairment, Risk stratification

## Abstract

**Background:**

Transbronchial lung cryobiopsy (TBLC) has become an established diagnostic modality in interstitial lung disease (ILD). However, current guidelines frequently exclude patients with severely impaired lung function due to safety concerns. This study aimed to evaluate the safety and diagnostic yield of TBLC in patients with advanced pulmonary function impairment.

**Methods:**

We conducted a retrospective analysis of consecutive patients with suspected ILD who underwent TBLC for diagnostic evaluation at Thoraxklinik Heidelberg, University Hospital Heidelberg, between November 2017 and December 2021. All cases were reviewed in a multidisciplinary discussion (MDD) before and after biopsy. Severe pulmonary function impairment was defined as a forced vital capacity (FVC) < 50% predicted and/or a diffusing capacity for carbon monoxide (DLCO_SB_) < 35% predicted. Procedural complications and diagnostic yield were assessed following post-procedural MDD.

**Results:**

A total of 396 patients were included (39% female, mean age 68 ± 12 years), of whom 43 patients (10.9%) had severely physiologically impaired ILD (SPI-ILD). The incidence of pneumothorax did not statistically differ between patients with severe and less physiologically impaired ILD (LPI-ILD) (11.6% vs. 14.7%, *p* = 0.818), with chest tube placement required in 33 cases overall. Severe bleeding necessitating endoscopic haemostasis occurred in 25.6% of SPI-ILD and 17.8% of LPI-ILD patients (*p* = 0.218). No surgical interventions for procedure-related complications were required. TBLC contributed to a final diagnosis following MDD review in 318 patients (80%), including 81% in SPI-ILD and 80% in LPI-ILD (*p* = 1.000).

**Conclusion:**

TBLC may represent a viable diagnostic option in carefully selected patients with severely impaired pulmonary function when performed in experienced centres following thorough risk–benefit assessment.

**Supplementary Information:**

The online version contains supplementary material available at https://doi.org/10.1186/s12931-026-03957-z.

## Introduction

Transbronchial lung cryobiopsy (TBLC) has become an established diagnostic tool for the evaluation of newly detected interstitial lung disease (ILD) of unknown cause and now plays an important role in the diagnostic work-up of ILD. Its value is supported by studies showing substantial agreement with surgical lung biopsy (SLB) for both histopathological interpretation and multidisciplinary discussion (MDD) diagnoses [1]. Current national and international recommendations advise that the indication for TBLC be determined within a MDD [2, 3]. However, TBLC is not suitable for all patients. Clinical studies, including the prospective multicentre COLDICE trial, excluded patients with a predicted DLCO < 40% [1]. Accordingly, current guidelines consider severe lung function derangement a relative contraindication for TBLC. The ATS/ERS/JRS/ALAT guideline on Idiopathic Pulmonary Fibrosis and Progressive Pulmonary Fibrosis defines severe impairment as DLCO < 35% predicted and/or FVC < 50% predicted, whereas the German national consensus guideline on the interdisciplinary diagnosis of ILD uses thresholds of DLCO-SB < 30% predicted and/or FVC < 50% predicted [2, 3]. At the same time, establishing a confident diagnosis is essential for guiding appropriate therapy, underscoring the need for careful risk–benefit assessment. Emerging evidence suggests that TBLC may still be a reasonable diagnostic option in selected high-risk patients who are not candidates for SLB because of impaired lung function or relevant comorbidities, particularly when performed in experienced, high-volume centres [2, 4–6]. In this context, we conducted a retrospective analysis at our centre for interventional endoscopy and interstitial and rare lung diseases to evaluate the complications and diagnostic outcomes of TBLC in patients with ILD of undetermined aetiology.

## Methods

### Study design and participants

This retrospective study included consecutive patients with suspected ILD referred to the Center for Interstitial and Rare Lung Diseases at Thoraxklinik Heidelberg, University Hospital Heidelberg, a tertiary referral centre. All patients who underwent TBLC between November 2017 and December 2021 were eligible for inclusion. Patients were identified through electronic medical records, and procedural complications were retrieved from bronchoscopy reports and corresponding clinical documentation.

At our institution, TBLC was performed within a standardized inpatient pathway. Patients were admitted on day 1 for preprocedural assessment, including pulmonary function testing; bronchoscopy and TBLC were performed on day 2. In the absence of clinical instability, patients were discharged on day 3. A chest radiograph was routinely obtained approximately 2 h after TBLC to screen for pneumothorax, and all patients remained under inpatient observation until discharge. Therefore, reported complications comprised events occurring during the procedure and throughout the subsequent inpatient observation period, which generally extended until the day after TBLC.

In cases of inconclusive histopathological findings, video-assisted thoracoscopic surgery (VATS) was offered after multidisciplinary discussion (MDD) for further diagnostic evaluation when patients were deemed eligible for surgery.

The study was approved by the Ethics Committee of the University of Heidelberg (S-382/2023) and conducted in accordance with the ethical standards of the Declaration of Helsinki, as amended in 2013. Given the retrospective design, the requirement for informed consent was waived.

### Pulmonary function testing

Pulmonary function testing was performed on the day of hospital admission, one day before bronchoscopy, using the Jaeger MasterScreen Body System (CareFusion, Rolle, Switzerland). Measurements included standardized bodyplethysmography and single-breath diffusing capacity for carbon monoxide (DLCO_SB_), conducted in accordance with American Thoracic Society (ATS) and European Respiratory Society (ERS) guidelines. Results were expressed as percent predicted values based on Global Lung Function Initiative (GLI) reference Eqs [7–11].

### Imaging

Computed tomography (CT) imaging was performed using a standardized non-contrast thin-section protocol with a slice thickness ≤ 1.5 mm (SOMATOM Definition AS, Siemens Healthineers AG) when no external imaging of comparable quality was available. All scans were acquired at full inspiration. CT datasets (reconstruction kernel i40f) were post-processed using the validated in-house software Yet Another CT Analyzer (YACTA, version 2.9.4.65), as previously described [12–15]. The software employs density-based thresholding to distinguish normal lung parenchyma from fibrotic tissue and incorporates morphological algorithms to segment the tracheobronchial tree, enabling separation of airway structures from surrounding parenchyma. All segmentations were visually reviewed for accuracy by an experienced reader. Quantitative analysis included extraction of Hounsfield unit (HU) values from the 10th to the 90th percentiles in 5% increments based on the histogram of lung voxel densities for each multidetector CT (MDCT) scan. In addition, the 40th and 80th percentile density values were specifically analysed, as these parameters have been previously associated with disease extent [13, 16].

### Bronchoscopy and periprocedural management

Bronchoscopy and periprocedural management were performed in accordance with institutional standards, as previously described [17]. Procedures were conducted under general anaesthesia using a combined rigid and flexible bronchoscopy approach. Biopsy targets were determined during the preceding MDD on the basis of clinical and radiological findings. When no specific sampling site was recommended, the performing bronchoscopist selected the target lobes and segments according to available imaging and procedural considerations. Samples were obtained from the peripheral lung parenchyma, maintaining a minimum distance of 1 cm from the pleura to ensure adequate representation, in line with expert recommendations [18]. A commercially available 1.7- or 1.9-mm cryoprobe (Erbe Elektromedizin GmbH, Tübingen, Germany) was used at the discretion of the bronchoscopist, with typical freezing times of 4–6 s. The intended sampling strategy comprised two cryobiopsies from each of two bronchopulmonary segments, preferably located in different lobes. This strategy represented a procedural target rather than a mandatory requirement; the number and location of biopsies could be modified intra-procedurally, and additional sampling could be omitted or the procedure terminated early in the event of clinically relevant bleeding or other safety concerns. Fluoroscopic guidance was not used uniformly throughout the study period. Overall, 32 bronchoscopists performed 396 procedures. Of these operators, 8.1% had less than 1 year of bronchoscopy experience, 27.8% had 1–3 years of experience, and 64.0% had more than 3 years of experience. Bronchoalveolar lavage and all cryobiopsy samples obtained during an individual procedure were performed by the same operator. The overall procedural approach to TBLC did not undergo a major change during the study period.

Anticoagulants and ADP-receptor antagonists were discontinued 48 h before bronchoscopy to reduce bleeding risk, whereas platelet aggregation inhibitors were continued. A chest radiograph was routinely obtained 2 h after the procedure to exclude pneumothorax, and all patients remained under inpatient observation for at least one night.

### Complication management and grading of bleeding events

Bleeding was managed in a stepwise manner according to the clinical situation. Because TBLC was performed under rigid bronchoscopy, neither prophylactic balloon inflation nor routine balloon occlusion after biopsy was used. Initial haemostatic management consisted of suction while maintaining the bronchoscope in a wedged position. If haemostasis was not achieved, temporary local tamponade with a swab was applied. For persistent bleeding requiring sustained local haemostasis, oxidised regenerated cellulose (Tabotamp^®^) was placed endobronchially and generally removed bronchoscopically after approximately 3 days. Bleeding severity was classified according to the haemostatic intervention documented in the procedural report. Bleeding controlled by bronchoscopic wedging and suction was classified as mild, whereas bleeding requiring endobronchial placement of Tabotamp^®^ was classified as severe. Bleeding requiring surgical intervention would have been classified as very severe; no such event occurred in this cohort.

Among patients who developed pneumothorax, the indication for chest-tube insertion was determined by the thoracic surgical team on the basis of clinical assessment rather than predefined study-specific criteria.

### Pathological evaluation

Histopathological evaluation was performed by an experienced pulmonary pathologist at the Department of Pathology, University Hospital Heidelberg. Specimen quality was categorized based on the pathology report into three groups: “good,” defined as representative samples with sufficient alveolar parenchyma; “moderate,” indicating limited or non-representative parenchyma; and “poor,” defined as absence of alveolar lung parenchyma.

### Multidisciplinary board discussion

MDD was conducted in two stages for each patient. The initial meeting evaluated the indication for bronchoscopic assessment, including transbronchial lung cryobiopsy, based on clinical data, imaging findings, and patient preferences. For the majority of the study period, national and international guidelines or position statements addressing contraindications to TBLC (e.g., impaired pulmonary function) were not yet available [3, 18–20]. The second meeting incorporated histopathological and bronchoalveolar lavage (BAL) results to establish a final diagnosis. In accordance with national and international guidelines, each MDD included at least two experienced pulmonologists, a thoracic radiologist and a thoracic pathologist [2, 3, 21]. A rheumatologist was involved when systemic disease was suspected.

### Categorization of ILD

Patients were categorized into four groups according to current German ILD guidelines [3, 22]. Group 1 comprised ILD of known cause (e.g., connective tissue disease–associated ILD, drug-induced ILD, post-radiation ILD). Group 2 included idiopathic ILD, predominantly idiopathic pulmonary fibrosis (IPF). Group 3 encompassed granulomatous ILD (hypersensitivity pneumonitis and sarcoidosis), while Group 4 included other ILD entities.

### Statistical analysis

Statistical analyses were performed using SPSS software (Version 31; IBM Corporation, Armonk, NY, USA). Data are presented as absolute numbers and percentages or as mean ± standard deviation (SD), unless otherwise specified. Categorical variables were compared using Fisher’s exact test, and continuous variables were analysed using two-tailed independent-samples t-tests.

For procedural safety outcomes, unadjusted absolute risk differences (RDs) were estimated using generalized linear models with a binomial distribution and identity link. RDs were defined as the event risk in the severe pulmonary function impairment group minus that in the less severely impaired group and are reported with 95% confidence intervals. Corresponding p-values were derived from Wald chi-square tests. A two-sided p value < 0.05 was considered statistically significant.

## Results

### Patient characteristics and ILD subgroups

A total of 396 patients, predominantly male (61%), with a mean age of 68 ± 12 years, were included. Patients were stratified into severely physiologically impaired ILD (SPI-ILD; FVC < 50% or DLCO_SB_ <35%, *n* = 43) and less physiologically impaired ILD (LPI-ILD, *n* = 353) cohorts based on ATS guidelines for IPF/PPF and a high-impact position paper [2, 18].

**Fig. 1:**
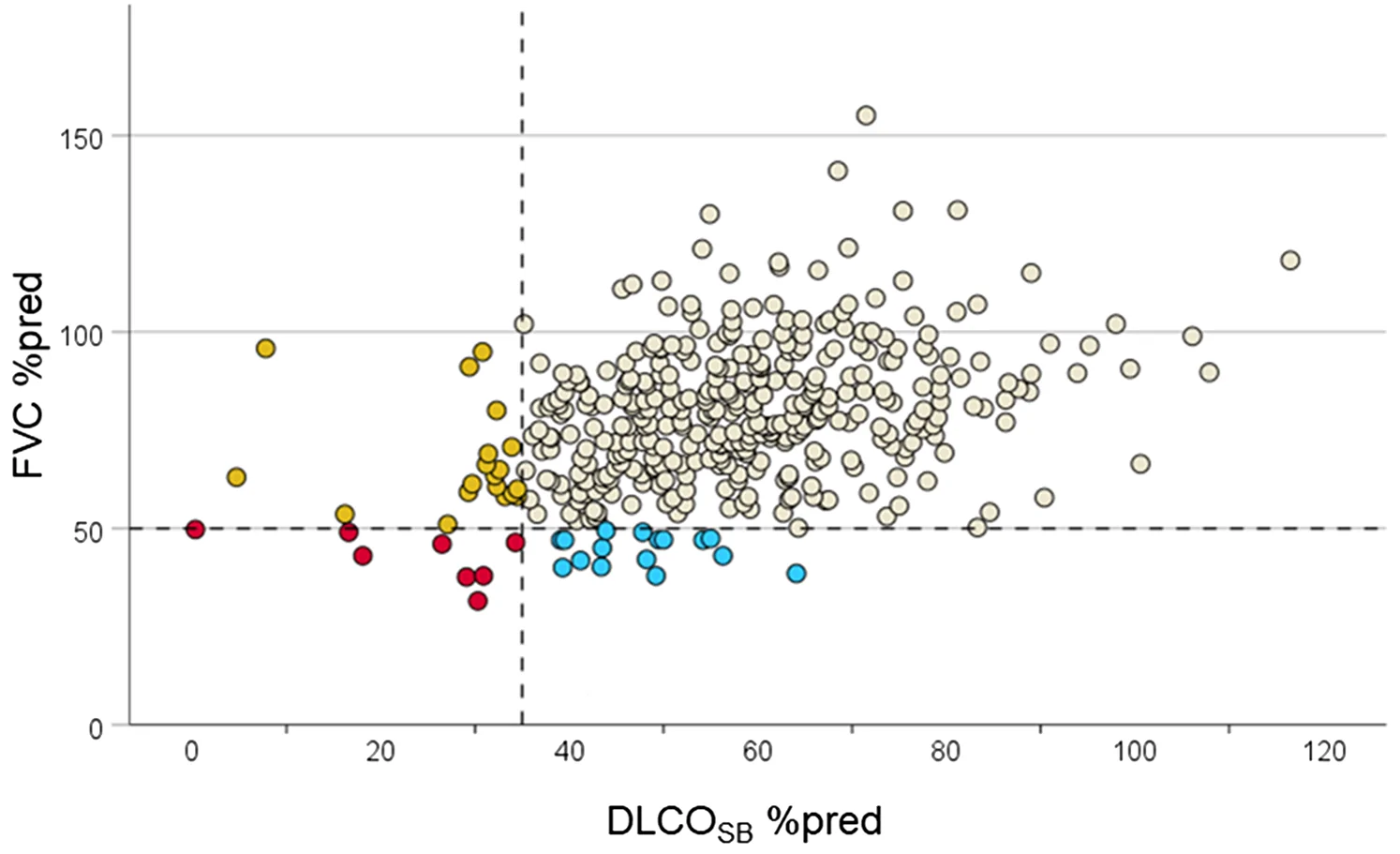
Study cohorts stratified by pulmonary function status (*n* = 396). Patients were stratified by forced vital capacity and single-breath diffusing capacity for carbon monoxide, both expressed as percent predicted. Overall, 353 patients (89.1%; brown dots) had less severe physiological impairment, whereas 43 patients (10.9%) met the predefined criteria for severe physiological impairment (FVC <50% predicted and/or DLCO-SB <35% predicted). Severe FVC impairment was observed in 24 patients (6.1%), including 16 patients (4.0%) with isolated FVC impairment. Severe DLCOSB impairment was present in 27 patients (6.8%), including 19 patients (4.8%) with isolated DLCOSB impairment. The overlap between these groups comprised 8 patients (2.0%) with both FVC <50% predicted and DLCOSB <35% predicted. Brown dots indicate patients without severe impairment according to the predefined thresholds; blue dots indicate isolated severe FVC impairment; yellow dots indicate isolated severe DLCOSB impairment; and red dots indicate combined severe impairment. Abbreviations: DLCOSB, diffusing capacity for carbon monoxide (single-breath). FVC: forced vital capacity

Among patients with SPI-ILD, 16 (37.2%) had isolated severe FVC impairment (FVC < 50% predicted and DLCO_SB_ ≥35% predicted), 19 (44.2%) had isolated severe DLCO_SB_ impairment (DLCO-SB < 35% predicted and FVC ≥ 50% predicted), and 8 (18.6%) had severe impairment in both measures (Fig. 1).

Baseline demographic characteristics, including age, sex, body mass index, smoking status, and the interval from symptom onset to diagnosis, were similar between groups. Although a history of smoking was similarly distributed, cumulative smoking exposure, expressed as pack years, was higher in the LPI-ILD group than in the SPI-ILD group (*p* = 0.026). The Charlson Comorbidity Index was also higher in the LPI-ILD group (*p* = 0.025). Conversely, heart failure was more frequent among patients with SPI-ILD than among those with LPI-ILD (2/43 vs. 1/353, *p* = 0.032). The prevalence of all other recorded comorbidities was comparable between groups (Table 1).

**Table 1:** Demographics and clinical characteristics

Variables	*n*	All mean ± SD or *n* (%) *n* = 396 (100.0)	Less physiologically impaired ILD mean ± SD or *n* (%) *n* = 353 (89.1)	Severely physiologically impaired ILD mean ± SD or *n* (%) *n* = 43 (10.9)	*p*
***Demographics***					
Age (years)	396	67.9 ± 11.7	68.2 ± 11.5	65.3 ± 13.5	0.177
**Proportion of females**	396	153 (38.6)	135 (38.2)	18 (41.9)	0.740
BMI (kg/m^2^)	396	28.7 ± 5.1	28.7 ± 5.3	28.8 ± 6.0	0.970
Interval from symptom onset to diagnosis (months)	396	19.5 ± 22.3	19.5 ± 22.6	19.2 ± 20.5	0.934
**Smokers**	396	239 (60.4)	217 (61.5)	22 (51.2)	0.247
Pack years	396	16.5 ± 21.9	17.2 ± 22.3	10.5 ± 17.8	**0.026**
***Pulmonary function***					
FEV_1_ (L)	396	2.4 ± 0.7	2.5 ± 0.7	1.8 ± 0.6	**< 0.001**
FEV_1_ (%predicted)	396	84.0 ± 20.6	86.7 ± 19.4	61.8 ± 17.0	**< 0.001**
FVC (L)	396	2.9 ± 0.9	3.0 ± 0.9	2.1 ± 0.7	**< 0.001**
FVC (%predicted)	396	77.5 ± 18.5	80.4 ± 16.8	54.2 ± 15.2	**< 0.001**
DLCO_SB_ (mmol/min/kPa)	396	4.8 ± 1.6	5.0 ± 1.5	3.2 ± 1.1	**< 0.001**
DLCO_SB_ (%predicted)	396	56.4 ± 16.2	59.1 ± 14.5	34.6 ± 13.4	**< 0.001**
***Laboratory parameters***					
Platelets (/nL)	396	238 ± 73	240.0 ± 72.8	218.4 ± 69.7	0.061
INR	394	1.03 ± 0.46	1.03 ± 05	1.0 ± 0.01	0.612
Quick (%)	394	100 ± 15	101.1 ± 15.1	99.0 ± 13.5	0.363
NTproBNP (ng/L)	228	197 ± 324	204.9 ± 338.6	133.9 ± 131.1	**0.051**
***Comorbidities***					
**Charlson Comorbidity Index**	396	3.5 ± 1.9	3.6 ± 1.9	3.0 ± 1.6	**0.025**
**Coronary Heart Disease**	396	58 (14.6)	54 (15.3)	4 (9.3)	0.367
**Peripheral artery disease**	396	10 (2.5)	9 (2.5)	1 (2.3)	1.000
**Stroke**	396	18 (4.5)	16 (4.5)	2 (4.7)	1.000
**Thromboembolism**	396	20 (5.1)	19 (5.4)	1 (2.3)	0.710
**Atrial Fibrillation**	396	26 (6.6)	22 (6.2)	4 (9.3)	0.508
**Heart Failure**	396	3 (0.8)	1 (0.3)	2 (4.7)	**0.032**
**Obstructive Sleep Apnoea**	396	51 (12.9)	49 (13.9)	2 (4.7)	0.096
**Chronic Kidney Disease**	396	22 (5.6)	22 (6.2)	0 (0.0)	0.151
**Diabetes mellitus type 2**	396	76 (19.2)	71 (20.1)	5 (11.6)	0.222
**Lung cancer**	395	3 (0.8)	3 (0.9)	0 (0.0)	1.000
**Gastroesophageal reflux disease**	396	195 (49.2)	176 (49.9)	19 (44.2)	0.521
***Imaging- Metrics***	350				
Lung volume (cm³)		4610 ± 1288	4724.2 ± 1270.5	3699.9 ± 1051.5	**< 0.001**
EI (%)		0.9 ± 1.9	0.9 ± 1.9	0.7 ± 2.3	0.674
FIBI (%)		29.4 ± 13.7	27.8 ± 12.7	41.3 ± 15.2	**< 0.001**
GGOI (%)		34.9 ± 16.1	34.6 ± 16.4	37.2 ± 13.4	0.302
80th percentile		-665 ± 91	-673.9 ± 90.1	-596.0 ± 71.5	**< 0.001**
40th percentile		-844 ± 43	-847.9 ± 41.5	-809.1 ± 41.3	**< 0.001**
**80th/40th percentile**		0.79 ± 0.09	0.8 ± 0.1	0.73 ± 0.1	**< 0.001**
***Imaging - Pattern***	396				
**UIP**		109 (27.5)	100 (28.3)	9 (20.9)	0.368
**NSIP**		203 (51.3)	179 (50.7)	24 (55.8)	0.628
**Consolidations**		92 (23.2)	78 (22.1)	14 (32.6)	0.129
**GGO**		242 (61.1)	214 (60.6)	28 (65.1)	0.622
**Honeycombing**		22 (5.6)	19 (5.4)	3 (7.0)	0.720
**Reticulations**		284 (71.7)	253 (71.7)	31 (72.1)	1.000

Severely physiologically impaired ILD was defined as FVC < 50% predicted and/or a DLCO_SB_ <35% predicted. Less physiologically impaired ILD was defined as FVC ≥ 50% predicted and/or a DLCO_SB_ ≥35% predicted

Abbreviations: BMI: Body Mass Index; DLCO_SB_: diffusing capacity of the lung for carbon monoxide, single breath method; EI: Emphysema Index; FEV1: forced expiratory volume in 1 s; FIBI: Fibrosis Index; FVC: forced vital capacity; GGOI: Ground‑Glass Opacity Index; ILD: interstitial lung disease; INR: International Normalized Ratio; NSIP: Non‑specific interstitial pneumonia; NTproBNP: N‑terminal pro‑B‑type natriuretic peptide; SD: Standard Deviation; UIP: usual interstitial pneumonia

**Table 2:** Procedural characteristics of bronchoscopies and transbronchial cryobiopsy

	*n*	Allmean±SD or *n* (%)*n*=396 (100.0)	Less physiologically impaired ILDmean±SD or *n* (%)*n*=353 (89.1)	Severely physiologically impaired ILDmean±SD or *n* (%)*n*=43 (10.9)	*p*
Number of samples	392	3.4±1.5	3.1±1.1	3.1±1.1	0.155
Sample size (mm)	393	6.2 ± 3.3	6.2 ± 3.5	6.1 ± 2.7	0.910
Duration (min)	394	27.8 ± 8.6	27.7 ± 8.6	28.5 ± 9.6	0.633
Use of fluoroscopy	396	257 (64.9)	228 (64.6)	29 (67.4)	0.866
Fluoroscopy duration (s)*	255	28.3 ± 20.1	28.9 ± 20.4	23.8 ± 17.0	0.145
Radiation dose (cGycm^2^)*	255	95.3 ± 80.3	95.8 ± 75.9	91.0 ± 110.2	0.819
Number of lobes	396	1.5 ± 0.5	1.5 ± 0.5	1.6 ± 0.5	0.438

Severely physiologically impaired ILD was defined as FVC < 50% predicted and/or a DLCO_SB_ <35% predicted. Less physiologically impaired ILD was defined as FVC ≥ 50% predicted and/or a DLCO_SB_ ≥35% predicted

Abbreviations: ILD: interstitial lung disease; SD: Standard Deviation

*Cases in which fluoroscopy was performed; in 2 patients no information on use of fluoroscopy was given

**Table 3:** Adverse Events and Outcome Measures of Bronchoscopies and Transbronchial Cryobiopsy

	*n*	All mean ± SD or *n* (%) *n* = 396 (100.0)	Less physiologically impaired ILD mean ± SD or *n* (%) *n* = 353 (89.1)	Severely physiologically impaired ILD mean ± SD or *n* (%) *n* = 43 (10.9)	*p*
**Complications**					
***Pneumothorax***	396	57 (14.4)	52 (14.7)	5 (11.6)	0.818
**No chest tube**		24 (6.1)	20 (5.7)	4 (9.3)	0.314
Chest tube		33 (8.3)	32 (9.1)	1 (2.3)	0.236
Surgery		0 (0.0)	0 (0.0)	0 (0.0)	-
***Bleeding*** *	396	136 (34.3)	121 (34.3)	15 (34.9)	1.000
Mild bleeding		62 (15.7)	58 (16.4)	4 (9.3)	0.272
Severe bleeding		74 (18.7)	63 (17.8)	11 (25.6)	0.218
**Outcomes**					
***Sample Quality*** ^**‡**^	396				
Good		239 (60.4)	219 (62.0)	20 (46.5)	0.068
Moderate		87 (22.0)	71 (20.1)	16 (37.2)	**0.018**
Poor		70 (17.7)	63 (17.8)	7 (16.3)	1.000
*BAL quality* ^*§*^	393				
Good		220 (56.0)	199 (56.9)	21 (48.8)	0.334
Moderate		82 (20.9)	72 (20.6)	10 (23.3)	0.692
Poor		91 (23.2)	79 (22.6)	12 (27.9)	0.445
Lymphocytosis (≥ 20%)	302	95 (31.5)	83 (30.6)	12 (38.7)	0.422
***MDD diagnosis***	396				
Diagnostic yield^#^		318 (80.3)	283 (80.2)	35 (81.4)	1.000
Definite diagnosis		240 (60.6)	211 (59.8)	29 (67.4)	0.409
High-probability diagnosis		45 (11.4)	41 (11.6)	4 (9.3)	0.802
Uncertain diagnosis		33 (8.3)	31 (8.8)	2 (4.7)	0.558
No diagnosis/VATS		78 (19.7)	70 (19.8)	8 (18.6)	1.000
VATS performed		30 (7.6)	28 (7.9)	2 (4.7)	0.758
***ILD subgroups***	396				
Group 1: Known causes		49 (12.4)	47 (13.3)	2 (4.7)	0.139
Group 2: Idiopathic		208 (52.5)	189 (53.5)	19 (44.2)	0.261
Group 3: Granulomatous ILD		118 (29.8)	96 (27.2)	22 (51.2)	**0.002**
Group 4: Others		11 (2.8)	11 (3.1)	0 (0.0)	0.617
No ILD		10 (2.5)	10 (4.0)	0 (0.0)	0.610

Severely physiologically impaired ILD was defined as FVC < 50% predicted and/or a DLCO_SB_ <35% predicted. Less physiologically impaired ILD was defined as FVC ≥ 50% predicted and/or a DLCO_SB_ ≥35% predicted

Abbreviations: BAL: bronchoalveolar lavage; ILD: interstitial lung disease; MDD: multidisciplinary discussion; SD: Standard Deviation; VATS: video‑assisted thoracoscopic surgery

^*^Severe bleeding was defined as bleeding requiring tamponade/balloon

^‡^Sample quality was categorized based on the pathology report with the following definitions applied: Good: Representative samples with sufficient alveolar parenchyma; Moderate: Limited or non-representative parenchyma; Poor: Absence of alveolar lung parenchyma

^§^The following definitions regarding BAL quality were applied: Good: Cell differentiation possible; Moderate: Cell differentiation with reservations; Poor: Cell differentiation not possible. In three cases, BAL was not performed. Percentages of lymphocytosis was given as percentage of evaluable BAL (*n* = 271 in less physiologically impaired ILD and *n* = 31 in severely physiologically impaired ILD)

^#^Diagnostic yield was defined as the percentage of cases where cryobiopsy contributed to a final diagnosis following MDD review

### Pulmonary function and imaging

By design, SPI-ILD patients exhibited significantly impaired pulmonary function (FEV_1_, FVC, DLCO_SB_; all *p* < 0.001). Haemostasis parameters were similar, but NT-proBNP levels were by trend higher in LPI-ILD (*p* = 0.051; *n* = 228) (Table 1). Figure 1 depicts the study cohort stratified by forced vital capacity (FVC, % predicted) and single-breath diffusing capacity for carbon monoxide (DLCO_SB_).

Quantitative CT metrics showed reduced lung volume in SPI-ILD (3700 ± 1052 cm³) compared to LPI-ILD (4724 ± 1271 cm³; *p* < 0.001), alongside higher fibrosis index (FIBI; SPI-ILD: 41.3 ± 15.2% vs. LPI-ILD: 27.8 ± 12.7%; *p* < 0.001) in SPI-ILD. The 80th/40th percentile lung density ratio was markedly lower in SPI-ILD (*p* < 0.001), consistent with greater ground-glass extent. No differences were observed in imaging patterns (UIP, NSIP) or radiological signs (ground-glass opacities, honeycombing, reticulations) between cohorts (Table 1).

### Bronchoscopy procedure characteristics

Cryobiopsies yielded a mean of 3.4 samples (size 6.2 ± 3.3 mm) from 1.5 lobes per procedure, with mean duration 27.8 ± 8.6 min. Fluoroscopy was used in 65% (mean radiation dose 95.3 ± 80.3 cGy·cm²). No intergroup differences were observed (Table 2).

### Complications

In-hospital pneumothorax occurred in 14.4% of patients overall, with chest tube requirement in 8.3%. No surgical interventions were needed. Pneumothorax occurred in 14.7% of patients in the LPI-ILD group and in 11.6% of those in the SPI-ILD group, corresponding to an absolute risk difference of − 3.1% points (95% CI, − 14.2 to 8.0; p = 0.584). Chest tube placement was required in 9.1% and 2.3% of patients in the LPI-ILD and SPI-ILD groups, respectively (Table 3 and Supplementary Table 2).

Bleeding occurred in 34.3% of patients overall, while severe bleeding requiring tamponade and/or balloon occlusion occurred in 18.7%. While overall bleeding did not differ between groups (LPI-ILD: 34.3% vs. SPI-ILD: 34.9%), severe bleeding was less frequent in LPI-ILD (17.8%) than SPI-ILD (25.6%) corresponding to an absolute risk difference of 7.7% points (95% CI, − 4.6 to 20.1; *p* = 0.218).

**Fig. 2:**
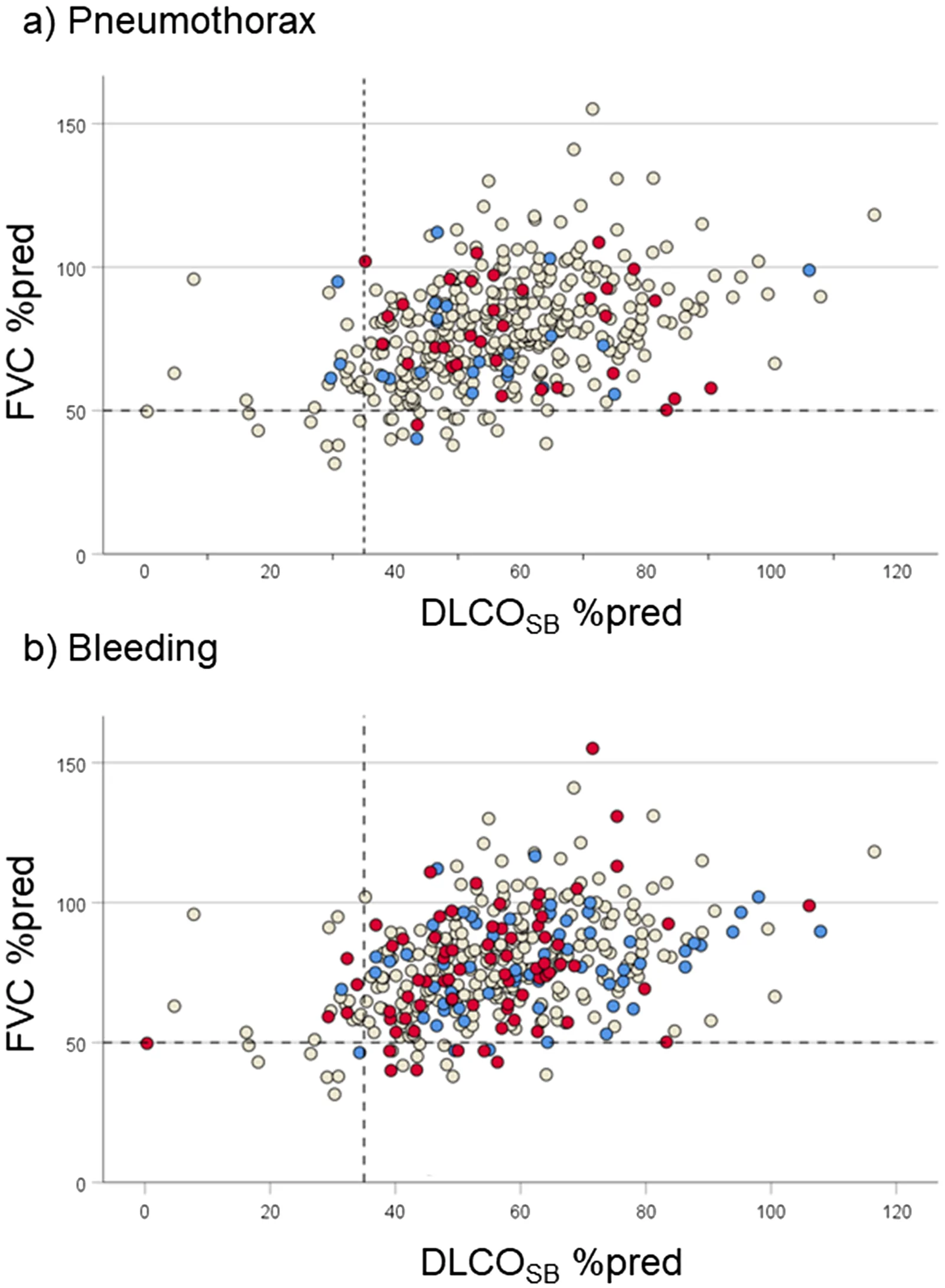
Pneumothorax and bleeding events following transbronchial lung cryobiopsy stratified by pulmonary function status (n=396). **a**) Pneumothorax: Brown dots indicate patients without pneumothorax (n = 339, 85.6%). Blue dots represent pneumothoraces not requiring chest tube placement (n = 24, 6.1%), whereas red dots indicate cases requiring chest tube insertion (n = 33, 8.3%). No patients required surgical intervention. Pneumothorax incidence did not differ significantly across pulmonary function strata. **b**) Bleeding: Brown dots indicate absence of bleeding (n = 260, 65.7%). Blue dots represent minor bleeding (n = 62, 15.7%), and red dots indicate severe bleeding requiring tamponade or balloon occlusion (n = 74, 18.7%). No surgical interventions or bleeding-related deaths occurred. Bleeding incidence was not significantly associated with pulmonary function status. Abbreviations: DLCOSB, diffusing capacity for carbon monoxide (single-breath). FVC: forced vital capacity

No in-hospital intensive care unit admissions or procedure-related deaths were observed. The distribution of pneumothorax and bleeding following TBLC, stratified by FVC % predicted and DLCOSB % predicted, is shown in Fig. 2. 

### Sample quality and diagnostic yield

Pathology-assessed tissue quality was good in 62% LPI-ILD vs. 47% SPI-ILD cases (p = 0.068), moderate in 20% LPI-ILD versus 37% SPI-ILD (p = 0.018), and poor in 18% LPI-ILD versus 16% SPI-ILD. BAL quality was good in 56% (cell differentiation possible), moderate in 21% (differentiation with reservations), and poor in 23% (differentiation not possible), with a nonsignificant trend favouring LPI-ILD (Table 3).

Diagnostic yield was defined as the percentage of cases where cryobiopsy contributed to a final diagnosis following MDD review. Overall diagnostic yield was 80% (61% definite, 11% high-probability, 8% uncertain diagnosis post-MDD). No diagnosis was reached in 20% and VATS was performed in 8%. No significant intergroup differences emerged, though a nonsignificant trend favoured definite diagnoses in SPI-ILD.

ILD subtypes were classified per German national guidelines [3, 22]. Most ILD were idiopathic (53%; predominantly IPF), followed by granulomatous ILD (30%), ILD of known-cause (12%), and other (3%). Granulomatous ILD was more prevalent in SPI-ILD (51% vs. 27%; *p* = 0.002). ILD diagnosis was withheld in 10 patients, all in LPI-ILD (Table 3).

## Discussion

This study evaluated the safety and diagnostic yield of TBLC in interstitial lung disease patients with severe pulmonary function impairment (FVC < 50% and/or DLCO_SB_ <35%). TBLC proved feasible with a tolerable safety profile even in this high-risk cohort, demonstrating no statistical difference in in-hospital pneumothorax (12% vs. 15%) and bleeding rates (35% vs. 34%) when compared to patients with less physiologically impaired ILD. Equally important, diagnostic yield was similar between groups, reaching 80% overall, thereby challenging conventional exclusion criteria based solely on impaired lung function [1, 18, 19, 21]. This aligns closely with prior ILD studies reporting ~ 79% yield [1, 2, 6, 17, 23–26]. Our results suggest that patients with severely impaired lung function should not automatically be excluded from diagnostic TBLC based solely of pulmonary function parameters. These results must be interpreted within the context of a single-centre study at an experienced tertiary referral centre with specialized expertise in interventional pulmonology and ILD diagnostics. Importantly, TBLC indications were established through structured multidisciplinary discussion, incorporating clinical history, comorbidities, laboratory data, imaging, and individual risk profiles.

To our knowledge, the present study represents one of the largest analyses to date specifically examining TBLC in patients with severe pulmonary impairment and further provides an in-depth characterization of this particularly vulnerable patient population. In these patients, procedural risk must against the potential consequences of an unclassified ILD, including delayed initiation of disease-specific treatment, uncertainty regarding prognosis, and missed opportunities for targeted therapeutic or supportive interventions. Additional strengths of our study include a standardized procedural protocol and the high level of expertise at a specialized ILD referral centre. The available evidence on the safety of TBLC in patients with advanced functional impairment remains limited and is largely derived from retrospective single-centre series or small multicentre cohorts with relatively few severely affected patients, resulting in heterogeneous findings [27, 28]. In a retrospective multicentre study from Australia, She et al. included 121 patients and identified severely reduced pulmonary function (DLCO_SB_ <35% or FVC < 50%) as a potential relative contraindication to TBLC [29]. However, despite 15 patients (12.4%) having a DLCO_SB_ <40%, no increase in complication rates was observed in this subgroup [29]. Similarly, Aburto et al. analysed 257 patients in a retrospective single-centre study assessing complications and 90-day hospital readmissions following TBLC in ILD patients [6]. Although multivariable analyses incorporated numerous clinical variables including FVC and DLCO_SB_, the limited number of events (39 complications, 23 readmissions) and retrospective design preclude definitive conclusions regarding the impact of severe impairment on procedural safety.

Comparable observations were reported in a prospective study by Bondue et al., which evaluated TBLC in patients considered high-risk based on predefined criteria, including severe pulmonary impairment (FVC < 50% predicted or DLCO_SB_ <30% predicted), advanced age (> 75 years), obesity (BMI > 35 kg/m^2^), pulmonary hypertension, or cardiac disease [4]. Among 96 patients, 38 were classified as high-risk; however, only 15 had severe pulmonary impairment. Consistent with our findings, complication rates—including pneumothorax, bleeding severity, and length of hospital stay—did not differ significantly between risk groups [4].

In a large cohort study of 699 patients, Ravaglia et al. identified a subgroup with marked functional impairment (FVC < 50% predicted and/or DLCO_SB_ <35% predicted; *n* = 31, 4% of the cohort) [5]. Despite the overall cohort size, the number of severely impaired patients was limited and lower than in our study, accounting for only approximately 4% of cases compared with ~ 11% in our study. In contrast to our results, both histopathological and MDD diagnostic yield were reduced in this subgroup, whereas complication rates remained comparable [5]. The lower diagnostic yield reported by Ravaglia et al. may partly reflect a more conservative procedural strategy in patients with advanced disease, including fewer and often unilobular biopsies [5]. In contrast, we applied standardized procedural parameters regarding sample number, size, and lobar distribution, irrespective of pulmonary function status. Although we noted a trend toward lower sample and BAL quality in our severely impaired group, this did not translate into a diminished final MDD diagnostic yield.

A recent case report further illustrates the potential diagnostic value of TBLC in patients with marked physiological impairment [30]. Poletti et al. reported the successful diagnosis of pulmonary capillary haemangiomatosis/pulmonary veno-occlusive disease by TBLC in a patient with severe diffusion impairment (DLCO_SB_ 16% predicted) and pulmonary hypertension. Although evidence from a single case cannot inform procedural safety, it underscores that marked functional impairment should not automatically exclude TBLC when the expected diagnostic benefit is substantial and the procedure is undertaken in an expert setting after careful multidisciplinary assessment.

An additional observation in this study was the higher prevalence of granulomatous ILD among patients with SPI-ILD. This category consisted predominantly of hypersensitivity pneumonitis, accounting for approximately 90% of cases. Although the retrospective design precludes causal inference, this distribution may reflect a subgroup of patients with fibrotic hypersensitivity pneumonitis and a progressive pulmonary fibrosis phenotype, in whom severe functional impairment may develop before a definitive diagnosis has been established.

Collectively, these data challenge the practice of excluding severely impaired ILD patients from TBLC based solely on pulmonary function thresholds. Instead, patients with ILD of undetermined aetiology and advanced pulmonary function impairment should undergo individualized risk stratification within a structured framework, including pulmonological assessment, anesthesiological evaluation, and multidisciplinary discussion, prior to proceeding with biopsy.

### Limitations

This study has several limitations. First, its retrospective design may introduce selection and reporting biases. In particular, patients who fulfilled the same physiological criteria but were discussed during MDD and ultimately not offered TBLC were not captured. Consequently, the SPI-ILD cohort reflects a clinically selected population considered suitable for bronchoscopy rather than the broader population of patients with severe physiological impairment. Second, the distribution of ILD subtypes differed between the SPI-ILD and LPI-ILD groups, potentially influencing both procedural risk and diagnostic yield. Third, bleeding severity was classified according to the haemostatic intervention required rather than a standardized quantitative bleeding scale, limiting direct comparability with studies applying alternative definitions. Fourth, only in-hospital complications were recorded. Because systematic post-discharge follow-up was not part of this retrospective study, delayed pneumothorax or other late procedure-related adverse events may have been missed. However, such events appear to be uncommon in our clinical experience. Most importantly, the SPI-ILD group was small and the number of safety events was limited. The study was therefore neither designed nor powered as an equivalence or non-inferiority analysis. Although no statistically significant between-group differences were observed, the confidence intervals surrounding the absolute risk differences were wide and remained compatible with clinically relevant increases or decreases in complication risk.

Finally, as a single-centre study conducted at a high-volume referral institution, the generalizability of our findings to centres with differing levels of expertise in ILD management and interventional pulmonology may be limited. Prospective multicentre studies are needed to validate these findings.

### Conclusion

In this selected cohort from an experienced tertiary referral centre, TBLC was not associated with an evident excess in procedure-related in-hospital complications among patients with severe pulmonary function impairment, while a high diagnostic yield could be maintained. However, the precision of these estimates was limited. Given that these patients may particularly benefit from an accurate diagnosis and timely initiation of targeted therapy, thorough diagnostic evaluation remains particularly important in this high-risk population. 

## Supplementary Information

Below is the link to the electronic supplementary material.

Supplementary Material 1

## Data Availability

The datasets used and/or analysed during the current study are available from the corresponding author on reasonable request.
