# Supplementary material for: Safety and diagnostic yield of transbronchial lung cryobiopsy in patients with interstitial lung disease and severe pulmonary function impairment
Source: Respir Res. 2026 Oct 13;27:406. doi: 10.1186/s12931-026-03957-z (PMC13673792; doi:10.1186/s12931-026-03957-z)
Supplement: Supplementary file 1 [file 12931_2026_3957_MOESM1_ESM.docx]

**Supplementary Table 1.** Complications and Diagnostic Outcomes Stratified by Pulmonary Function Phenotypes in Patients with Severely Physiologically Impaired Interstitial Lung Disease (n=43).

|  | ILD with isolated FVC impairment  mean±SD or n (%)  n=16 (37.2) | ILD with isolated DLCO_SB_ impairment  mean±SD or n (%)  n=19 (44.2) | ILD with combined FVC/DLCO_SB_ impairment  mean±SD or n (%)  n=8 (18.6) |
| --- | --- | --- | --- |
| Complications |  |  |  |
| *Pneumothorax* | 2 (12.5) | 3 (15.8) | 0 (0.0) |
| No chest tube | 1 (6.3) | 3 (15.8) | 0 (0.0) |
| Chest tube | 1 (6.3) | 0 (0.0) | 0 (0.0) |
| Surgery | 0 (0.0) | 0 (0.0) | 0 (0.0) |
|  |  |  |  |
| *Bleeding* * | 8 (50.0) | 5 (26.3) | 2 (25.0) |
| Mild bleeding | 2 (12.5) | 1 (5.3) | 1 (5.3) |
| Severe bleeding | 6 (37.5) | 4 (21.1) | 1 (12.5) |
|  |  |  |  |
| Outcomes |  |  |  |
| *Sample Quality* ^‡^ |  |  |  |
| Good | 9 (56.3) | 11 (57.9) | 0 (0.0) |
| Moderate | 4 (25.0) | 6 (31.6) | 6 (75.0) |
| Poor | 3 (18.8) | 2 (10.5) | 2 (25.0) |
| *BAL quality ^§^* |  |  |  |
| Good | 7 (43.8) | 11 (57.9) | 3 (37.5) |
| Moderate | 7 (43.8) | 3 (15.8) | 0 (0.0) |
| Poor | 2 (12.5) | 5 (26.3) | 5 (62.5) |
| Lymphocytosis (≥ 20%) *^§^* | 4 (28.6) | 7 (50.0%) | 1 (33.3) |
|  |  |  |  |
| *MDD diagnosis* |  |  |  |
| Diagnostic yield ^#^ | 16 (100.0) | 14 (73.7) | 5 (62.5) |
| Definite diagnosis | 14 (87.5) | 12 (63.2) | 3 (37.5) |
| High-probability diagnosis | 1 (6.3) | 1 (5.3) | 2 (25.0) |
| Uncertain diagnosis | 1 (6.3) | 1 (5.3) | 0 (0.0) |
| No diagnosis/VATS | 0 (0.0) | 5 (26.3) | 3 (37.5) |
| VATS performed | 0 (0.0) | 2 (10.5) | 0 (0.0) |
|  |  |  |  |
| *ILD subgroups* |  |  |  |
| Group 1: Known causes | 1 (6.3) | 0 (0.0) | 1 (12.5) |
| Group 2: Idiopathic | 6 (37.5) | 8 (42.1) | 5 (62.5) |
| Group 3: Granulomatous ILD | 9 (56.3) | 11 (57.9) | 2 (25.0) |
| Group 4: Others | 0 (0.0) | 0 (0.0) | 0 (0.0) |
| No ILD | 0 (0.0) | 0 (0.0) | 0 (0.0) |

ILD with isolated FVC impairment was defined as FVC <50% and DLCOSB ≥35%, ILD with isolated DLCO_SB_ impairment was defined as DLCO_SB_ <35 and FVC ≥50% and ILD with combined FVC/DLCO_SB_ impairment was defined as FVC <50% and DLCO_SB_ <35%.

**Abbreviations:** BAL: bronchoalveolar lavage**;** DLCO_SB_: diffusing capacity of the lung for carbon monoxide, single breath method (% predicted); FVC: forced vital capacity (% predicted); ILD: interstitial lung disease; MDD: multidisciplinary discussion; SD: Standard Deviation; VATS: video‑assisted thoracoscopic surgery.

* Severe bleeding was defined as bleeding requiring tamponade/balloon

‡ Sample quality was categorized based on the pathology report with the following definitions applied: Good: Representative samples with sufficient alveolar parenchyma; Moderate: Limited or non-representative parenchyma; Poor: Absence of alveolar lung parenchyma.

§ The following definitions regarding BAL quality were applied: Good: Cell differentiation possible; Moderate: Cell differentiation with reservations; Poor: Cell differentiation not possible.

n=31. 14 BAL were evaluable in ILD with isolated FVC impairment, 14 in ILD with isolated DLCO_SB_ impairment and 3 in ILD with combined FVC/DLCO_SB_ impairment.

^#^ Diagnostic yield was defined as the percentage of cases where cryobiopsy contributed to a final diagnosis following MDD review.

**Supplementary Table 2.** Absolute Risk Differences and 95% Confidence Intervals for Procedural Complications following Transbronchial Lung Cryobiopsy (n=396).

| Variables | Severely physiologically impaired ILD  n (%)  n=43 (10.9) | Less physiologically impaired ILD  n (%)  n=353 (89.1) | Absolute Risk Difference (%) * | 95% CI | p ^‡^ |
| --- | --- | --- | --- | --- | --- |
| Pneumothorax – Overall | 5 (11.6) | 52 (14.7) | -3.1 | -14.2 to 8.0 | 0.584 |
| Pneumothorax with Chest tube | 1 (2.3) | 32 (9.1) | -6.7 | -15.5 to 2.0 | 0.130 |
| Bleeding – Overall | 15 (34.9) | 121 (34.3) | +0.6 | -14.4 to 15.6 | 0.937 |
| Severe Bleeding | 11 (25.6) | 63 (17.8) | +7.7 | -4.6 to 20.1 | 0.218 |

Severely physiologically impaired ILD was defined as FVC <50% predicted and/or a DLCO_SB_ <35% predicted. Less physiologically impaired ILD was defined as FVC ≥50% predicted and/or a DLCO_SB_ ≥35% predicted.

**Abbreviations:** CI: confidence interval; ILD: interstitial lung disease.

* Calculated as the absolute risk in the severely physiologically impaired ILD group minus the risk in the less physiologically impaired ILD group.

p-values were calculated using the Wald chi-square test within the generalized linear model (GZLM) framework

^‡^ p-values were calculated using the Wald chi-square test within the generalized linear model framework.
